# Hypofractionated radiotherapy plus PD-1 antibody and SOX chemotherapy as second-line therapy in metastatic pancreatic cancer: a single-arm, phase II clinical trial

**DOI:** 10.1007/s00262-024-03744-z

**Published:** 2024-08-06

**Authors:** Qin Wang, Fan Tong, Li Qiao, Liang Qi, Yi Sun, Yahui Zhu, Jiayao Ni, Juan Liu, Weiwei Kong, Baorui Liu, Juan Du

**Affiliations:** 1 Department of Oncology, Nanjing Drum Tower Hospital, Affiliated Hospital of Medical School, Nanjing University & Clinical Cancer Institute of Nanjing University, Nanjing, 210008 China; 2 Department of Oncology, Nanjing Drum Tower Hospital, Clinical College of Nanjing Drum Tower Hospital, Nanjing University of Chinese Medicine, Nanjing, 210008 China; 3 Comprehensive Cancer Center of Drum Tower Hospital, Medical School of Nanjing, University&Clinical Cancer Institute of Nanjing University, 321 Zhongshan Road, Nanjing, 210008 China

**Keywords:** Metastatic pancreatic cancer, Immune checkpoint inhibitors, Hypofractionated radiotherapy, SOX chemotherapy, Second-line therapy

## Abstract

**Purpose:**

To assess the efficacy and safety of concurrent hypofractionated radiotherapy plus anti-PD-1 antibody and SOX chemotherapy in the treatment of metastatic pancreatic cancer (mPC) after failure of first-line chemotherapy.

**Methods:**

Patients with pathologically confirmed mPC who failed standard first-line chemotherapy were enrolled. The patients were treated with a regimen of hypofractionated radiotherapy, SOX chemotherapy, and immune checkpoint inhibitors at our institution. We collected the patients’ clinical information and outcome measurements. The median progression-free survival (mPFS) was the primary endpoint of the study, followed by disease control rate (DCR), objective response rate (ORR), median overall survival (mOS) and safety. Exploratory analyses included biomarkers related to the benefits.

**Results:**

Between February 24, 2021, and August 30, 2023, twenty-five patients were enrolled in the study, and twenty-three patients who received at least one dose of the study agent had objective efficacy evaluation. The mPFS was 5.48 months, the mOS was 6.57 months, and the DCR and ORR were 69.5% and 30.4%, respectively. Among the seven patients who achieved a PR, the median duration of the response was 7.41 months. On-treatment decreased serum CA19-9 levels were associated with better overall survival. Besides, pretreatment inflammatory markers were associated with tumor response and survival.

**Conclusions:**

Clinically meaningful antitumor activity and favorable safety profiles were demonstrated after treatment with these combination therapies in patients with refractory mPC. On-treatment decreased serum CA19-9 levels and pretreatment inflammatory markers platelet-to-lymphocyte ratio (PLR), lymphocyte-to-monocyte ratio (LMR), lactate dehydrogenase (LDH) might be biomarkers related to clinical benefits.

*Clinical trial registration*: https://www.chictr.org.cn/showproj.html?proj=130211, identifier: ChiCTR2100049799, date of registration: 2021–08-09.

**Supplementary Information:**

The online version contains supplementary material available at 10.1007/s00262-024-03744-z.

## Introduction

Pancreatic ductal adenocarcinoma (PDAC) is one of the deadliest solid tumors, and the 5-year survival rate is the lowest among various cancers (9% for all stages) [1]. Due to the tendency for late diagnosis, early metastatic nature, aggressive local invasion, and resistance to systemic therapy [2, 3], the prognosis of patients with PDAC remains dismal. Even in patients undergoing curative resection, survival remains poor, with a 5-year survival rate less than 20% and a 5-year overall survival (OS) lower than 7% [4]. Based on the result of phase 3 NAPOLI-1 trial, nanoliposomal irinotecan/5-FU/leucovorin is the only approved second-line treatment for patients suffering from metastatic pancreatic ductal adenocarcinoma (mPDAC) [5]. The median OS duration for patients treated with nal-IRI + 5-FU/LV was 6.1 months, compared to 4.2 months for those receiving 5-FU/LV. Additionally, the overall response rate was 16% in patients treated with nal-IRI + 5-FU/LV, whereas it was only 1% in those receiving 5-FU/LV. However, the combination chemotherapy group had a higher occurrence of grade 3/4 adverse reactions. Additionally, the patients receiving second-line treatment for advanced pancreatic cancer have such poor physical condition that they may be unable to tolerate this combined chemotherapy regimen. 5-Fu-based chemotherapy or capecitabine-based chemotherapy are also used after progression under gemcitabine-based therapy [6]. S-1 monotherapy in gemcitabine-refractory metastatic pancreatic cancer has been reported to show some efficacy in a previous phase II trial, the objective response rate (ORR) was 4.7%, overall survival (OS) was 5.5 months, and most of those adverse reactions were tolerable [7]. In Japan, S-1 is commonly used for the treatment of gemcitabine-refractory metastatic pancreatic cancer. In the phase II study of S-1 for pancreatic cancer resistant to GEM, the objective response rate (ORR) was 15%, median progression-free survival (PFS), and overall survival (OS) were only 2.0 months and 4.5 months, respectively, and most of those adverse reactions were tolerable [8]. Whereas, no regimen has demonstrated superiority in the second-line, and there is urgent need for innovative therapies to address the challenges associated with this disease.

Immunotherapy represents a promising development in the treatment of cancer; conversely, the results of investigations into its efficacy as immunotherapy alone for PDAC have mostly been disappointing [9–11]. Immunotherapy can achieve clinical benefits in only a small subset of PC patients displaying microsatellite instability (MSI) or mismatch repair (MMR) deficiency [12]. This may be attributed to the low TMB and poor microenvironment of pancreatic cancer. Preclinical studies have indicated that chemotherapy can induce immunogenic apoptosis of tumor cells, enhancing T-cell infiltration, and reactivity [13–15]. Thus, combining chemotherapy and immune checkpoint inhibitors (ICIs) may enhance the immune response against tumors in pancreatic cancer. However, the results of these combination therapies were disappointing. Wainberg et al. [16] reported that the combination of nivolumab and gemcitabine or nab-paclitaxel did not improve the response rate of patients with advanced pancreatic cancer, while Weiss et al. [17] reported a slightly improved response rate to chemoimmunotherapy in patients with metastatic pancreatic cancer. Therefore, studies combining ICIs with novel therapeutics to improve clinical treatment efficacy are emerging in preclinical and clinical settings [18–21].

Radiotherapy (RT) has a long history in the treatment of tumors. RT has been reported to be a beneficial treatment for many types of cancers [22, 23] and can improve the local control rate and delay postoperative recurrence. Although the use of RT alone for pancreatic cancer treatment is rare due to the spatial location of the cancer, the benefits of RT alone or combined with chemotherapy for pancreatic cancer treatment are still under debate [23–25]. Therefore, the combination of RT and ICIs may provide a rational strategy for pancreatic cancer treatment. Preclinical studies have shown that the addition of RT to immunotherapy can have a synergistic effect and enhance antitumor activity [26–28]. It is hypothesized that local RT can induce immunological death of tumors, increase the presentation of neoantigens within the tumor stroma and improve immune cell infiltration, thereby stimulating antitumor immunity [29].

In recent clinical trials, the combination of immunotherapy with stereotactic body radiotherapy (SBRT) has demonstrated clinically meaningful antitumor activity and favorable safety profiles [30, 31]. This combination has resulted in a disease control rate of up to 30% in patients with refractory PDAC. A phase II study conducted by our team revealed that PD-1 blockage combined with chemoradiotherapy as a preoperative therapy is potentially effective, resulting in a high ORR and an outstanding R0 resection rate without serious adverse reactions or postoperative complications [32]. Based on successful regimens and promising clinical trials, we conducted this trial. Our objective was to investigate the safety and effectiveness of combining PD-1 blockade with chemoradiotherapy as a second-line therapy for patients with metastatic pancreatic cancer who did not respond to first-line gemcitabine chemotherapy.

## Methods

### Patient population

**Fig. 1:**
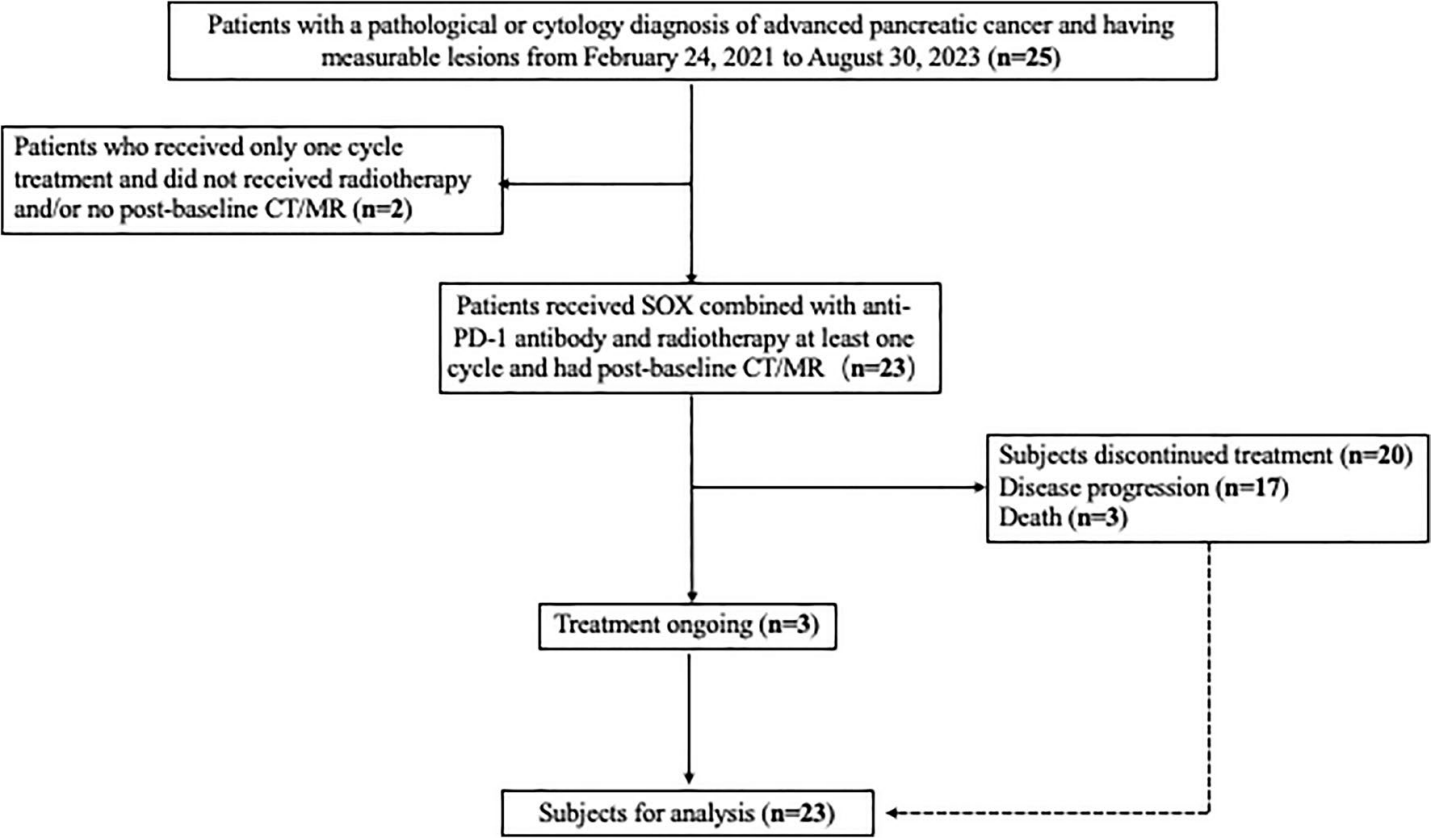
Flow diagram of the study population. Abbreviations: *CT*, computed tomography; *MRI*, magnetic resonance imaging

This was a single-arm, open-label, phase II study to determine the safety and efficacy of SOX/anti-PD-1 antibody/hypofractionation radiotherapy in patients with gemcitabine-refractory mPC from February 24, 2021 to August 30, 2023, at the Comprehensive Cancer Centre of Drum Tower Hospital, Clinical Cancer Institute of Nanjing University. Prior to therapy, written informed consent was obtained from all patients. The ethical committee of Nanjing Drum Tower Hospital granted approval for this study. Flow diagram of the study population is shown in Fig. 1. The details of the inclusion and exclusion criteria are presented in Table S1.

### Treatment

Patients received S-1 (25 mg/m^2^) orally twice/day for 2 weeks, followed by 1 week of rest, intravenous oxaliplatin (130 mg/m^2^) on day 1 of each 3-week cycle and anti-PD-1 antibody (sintilimab, toripalimab) intravenously (200 mg and 240 mg, respectively) on day 1 of each 3-week cycle. Concurrent radiotherapy was administered to patients after the completion of one cycle of systemic therapy. Computerized tomography (CT) simulation was conducted approximately one week after the first cycle of chemotherapy plus immunotherapy, and a gross target volume (GTV) was generated based on the complete extent of one to three distant metastases per the researchers’ and radiation therapy technologists’ discretion as delineated in each CT phase. A clinical target volume (CTV) was then generated, with a 0.5 cm expansion around the GTV. The planning organ at risk volume (PRV) was then defined as a 1-cm expansion of the gastrointestinal tract, and the planned gross tumor volume (PGTV) was defined as a 0.3-cm expansion from the GTV, with the PRV subtracted. The PTV was defined as a 0.5-cm expansion around the CTV. SBRT was administered daily, with a total dose of 24 Gy/3f delivered at the PGTV and 15 Gy/3f delivered at the PTV concurrently with the second cycle of chemotherapy and immunotherapy.

Treatment interruptions, dose reductions, and supportive care were allowed for effective management of any adverse events (AEs) experienced by patients. Treatment was continued until the occurrence of progressive disease (PD), intolerable toxicity, withdrawal of consent, or any other factor necessitating discontinuation of treatment. In patients who experienced grade 3 or higher AEs, treatment was suspended, and AEs were treated until they disappeared or reached grade 1 or 2. If patient experienced grade III hematological toxicity, the chemotherapy dosage is reduced by 20%. For patients experiencing PD, survival data were tracked until completion of the study. For those who discontinued treatment for other reasons, tumor response and safety profiles were evaluated every 2 months until disease progression or completion of the study.

### Assessment

The assessment of tumor response via CT scans was carried out according to the RECIST v.1.1 guidelines. This assessment was conducted at baseline and after every two cycles of treatment. The protocol necessitated the measurement of peripheral blood biomarkers at both baseline and after every two cycles of treatment. Blood samples were analyzed for peripheral neutrophil, lymphocyte, NK cell, peripheral blood eosinophil count (PBEC), neutrophil- to-lymphocyte ratio (NLR), platelet-to-lymphocyte ratio (PLR), lymphocyte-to-monocyte ratio (LMR), lactate dehydrogenase (LDH) as well as carbohydrate antigen 19–9 (CA19-9) levels. Patients who had normal CA19-9 levels (< 27 U/mL) at baseline were excluded from the CA19-9 response assessment because they were less likely to display a significant decrease in CA19-9 levels. Furthermore, if the CA19-9 concentration exceeded 27, the response was evaluated. Moreover, immunostaining of PD-L1 with VENTANA PD-L1 (SP263) antibody in tumor biopsies were also performed in 7 patients. PD-L1 positive was defined as tumor cell positivity rate (TC +) higher than 1%. All AEs were recorded and rated based on the National Cancer Institute Common Terminology Criteria (NCI-CTCAE) version 5.0. The AEs were graded for severity and evaluated at all patient visits from baseline to the short-term follow-up.

### Endpoints

The primary outcome of the trial was progression-free survival (PFS). PFS was defined as survival without any progressive disease from the date of enrollment. The secondary endpoints were safety, median overall survival (OS), disease control rate (DCR), and objective response rate (ORR). OS was defined as the duration from the date of enrollment to the date of death from any cause. The DCR refers to the percentage of patients with remission and stable disease among the population. The ORR is defined as the proportion of patients who achieve a complete response (CR) or partial response (PR) based on the Response Evaluation Criteria in Solid Tumors (RECIST). The exploratory endpoints included analyses of biomarkers associated with clinical efficacy outcomes.

### Statistical analysis

The study is an open-label, single-arm, phase II clinical trial with plans to enroll 25 eligible participants. The sample size was not determined based on statistical hypotheses. The baseline demographic and clinical characteristics will be presented using descriptive analyses such as tables and charts. Statistical analysis was conducted using SAS statistical software version 9.4 by the SAS Institute. In the intention-to-treat population, efficacy and safety outcome analyses were performed on patients who underwent one or more posttreatment scans. Categorical variables, including patients with objective response or adverse events, will be summarized by descriptive statistical analysis with a 95% confidence interval using the Wilson score method. Continuous variables will be expressed as the median (range). Fisher's exact test was employed to assess response differences (ORRs) and other binary outcomes among the clinical subgroups. Furthermore, we generated Kaplan‒Meier plots for PFS and OS, and the log-rank test was used to compare the survival functions among different subgroups. Exploratory univariate analyses were conducted with the log-rank test using the following variables: age, sex, ECOG score, CA19-9, and number of metastatic sites. Responses and adverse events were both aggregated as frequency counts and percentages. The optimal cutoff values for PLR, LMR, and LDH were 125, 2.5, and 200, respectively, and were determined using R Foundation (version 4.0.1) and SPSS software (version 26.0). For all analyses, a p value less than 0.05 was considered indicative of statistical significance.

## Results

### Baseline demographic and clinical characteristics

From February 24, 2021, to August 30, 2023, a cohort of 25 patients diagnosed with mPC was enrolled in our study. Of these, 23 patients included in the intention-to-treat (ITT) analysis received at least one cycle of PD-1 plus SOX therapy and concurrent radiotherapy until their last follow-up on August 30, 2023. The majority of patients (*n* = 12, 52.2%) were female, with an average age of 65.3 (IQR 53–80) years and an Eastern Cooperative Oncology Group Performance Status (ECOG PS) of 0 (*n* = 6, 26.1%), 1 (*n* = 15, 65.2%), or 2 (*n* = 2, 8.7%). The baseline demographics and clinical characteristics of the patients at treatment initiation are presented in Table 1**.** This section summarizes the key features and patient recruitment status of our research.

**Table 1:** Baseline patient and demographic and disease characteristics

Characteristic	N = 23
Age	
Mean (SD)	65.3 (8.00)
Median [Min, Max]	66.0 [53.0, 80.0]
Gender	
Female	12 (52.2%)
Male	11 (47.8%)
BMI	
Mean (SD)	21.7 (2.66)
Median [Min, Max]	21.6 [16.0, 27.6]
Primary site of cancer	
Body	4 (17.4%)
Body-tail	8 (34.8%)
Head	8 (34.8%)
Tail	3 (13.0%)
Surgery	
NO	20 (87.0%)
YES	3 (13.0%)
Duration day of first-line treatment	
Mean (SD)	204 (201)
Median [Min, Max]	157 [34.0, 1060]
Radiotherapy site	
Liver	12 (52.2%)
Not Liver	11 (47.8%)
Number of distant metastases	
Mean (SD)	1.61 (1.08)
Median [Min, Max]	1.00 [0, 4.00]
BaselineCA199	
Mean (SD)	3450 (8760)
Median [Min, Max]	445 [0.600, 41700]
BestefficacyCA199	
Mean (SD)	2570 (3470)
Median [Min, Max]	577 [2.00, 11700]
ChangeOfCA199	
Mean (SD)	-873 (8050)
Median [Min, Max]	-0.0600 [-36200, 6670]
ΔCA199	
Decrease	12 (52.2%)
Increase	11 (47.8%)

### Efficacy

**Fig. 2:**
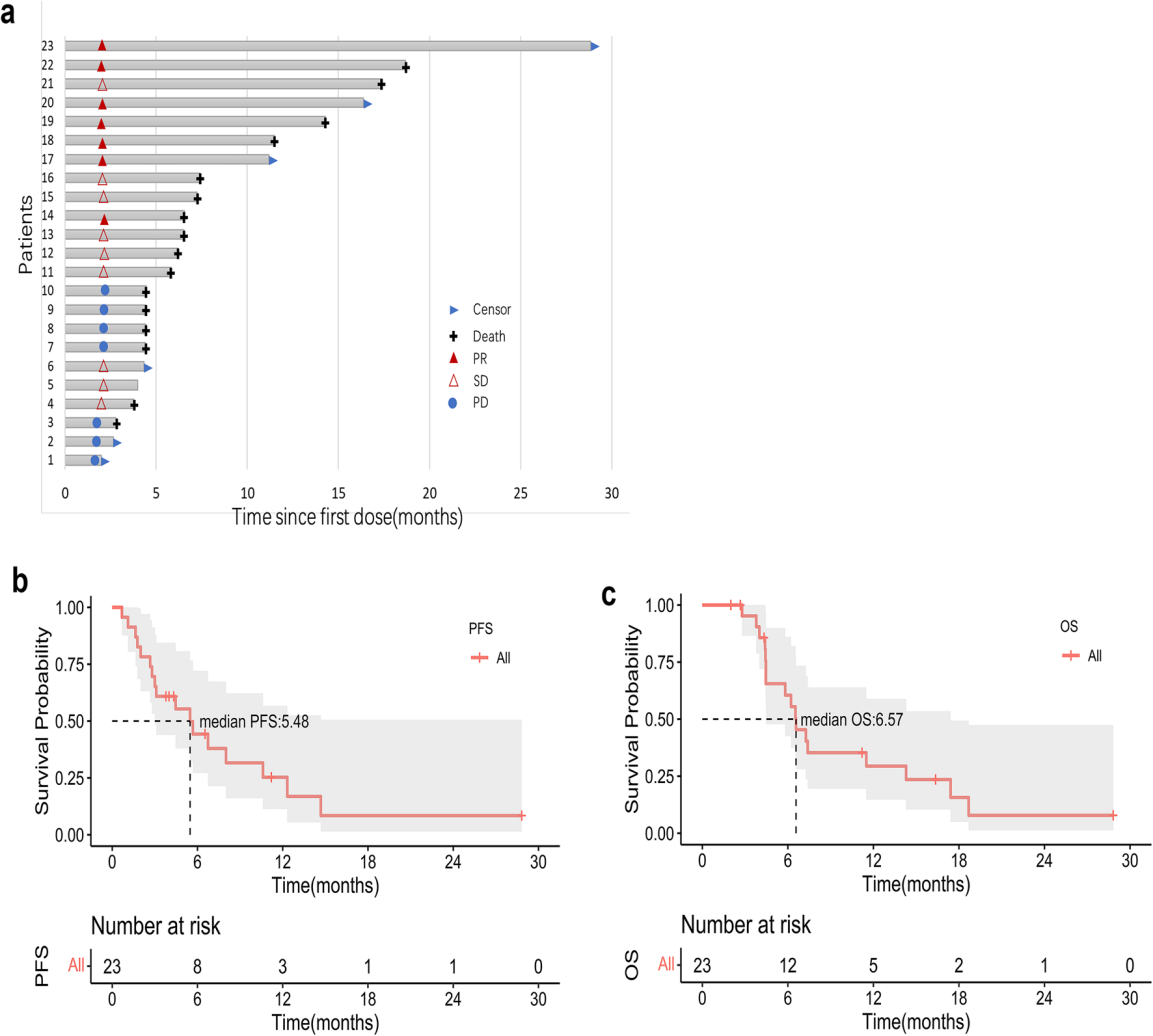
Treatment response and survival analysis **A** Duration of responses of patients in the ITT population. The length of each bar represents the duration of treatment of each patient. **B** and **C** The Kaplan–Meier curves of **B** PFS and **C** OS in all enrolled patients

A total of 23 patients were included in the final analysis. At the time of data cutoff, 7 patients achieved a PR, 9 patients achieved stable disease (SD), and 7 patients had progressive disease (PD). The ORR was 30.43%, and the DCR was 69.57% (Table 2). The overall treatment results are presented using swimmer charts in Fig. 2a. Notably, four exceptional responders had a continuing response after inclusion and are still alive. Regarding survival outcomes, the median PFS and OS were 5.48 (95% CI, 3 to not reached) and 6.57 months (95% CI, 4.46 to 17.4), respectively. The Kaplan‒Meier analysis of PFS and OS is shown in Fig. 2b**‒c**.

**Table 2:** Statistical associations between baseline NLR, PLR, LMR, LDH, and tumor response

		NLR N (%)			PRL N (%)			LMR N (%)			LDH N (%)		
Total(N = 23) N(%)		≤ 3	> 3	P-value	< 125	≥ 125	P-value	≤ 2.5	> 2.5	P-value	≤ 200	> 200	P-value
Best overall response													
CR	0(0)	0(0)	0(0)		0(0)	0(0)		0(0)	0(0)		0(0)	0(0)	
PR	7(30.43)	5 (71.43)	2 (28.57)		6(85.71)	1(14.29)		7(100)	0(0)		1(14.28)	6(85.71)	
SD	9(39.14)	7 (77.78)	2 (22.22)		2(22.22)	7(77.78)		4(44.44)	5(55.56)		3(33.33)	6(66.64)	
PD	7(30.43)	3 (42.85)	4 (57.15)		3(42.85)	4(57.15)		2(28.57)	5(71.43)		6(85.71)	1(14.29)	
DCR(CR + PR + SD)	69.57	12 (80)	4 (50%)	0.1819	72.72	66.67	1	84.61	50	0.1688	40	92.3	**0.0186**
ORR(CR + PR)	30.43	5 (33.3)	2 (25%)	1	54.55%	8.33%	**0.0272**	53.84	0	**0.0075**	10	46.15	0.0886

P values in bold indicates the statistically significant differences (P < 0.05), which indicate better results than other groups

### Safety

Hematological and nonhematological toxicities during our treatment are summarized in Table 3. No patients died of treatment-related adverse events (TRAEs). A total of 23 patients experienced TRAEs of any grade, possibly due to the poor physical condition of the patient after the failure of first-line treatment. Among them, 7 patients had grade 3/4 TRAEs. The most commonly observed grade 3/4 TRAEs included lymphocytopenia (22%), thrombocytopenia (17%), anemia (13%), increased aspartate transaminase (AST) levels (13%), leukocytopenia (9%), neutropenia (9%), hypopotassemia (4%), toxic epidermal necrolysis (4%), and vomiting (4%). None of the patients experienced any serious immune-related AEs, such as autoimmune myocarditis or pneumonitis.

**Table 3:** Summary of adverse events by severity

Adverse events	Any grade, n (%)	Grades 3–4, n (%)	Treatment-related grade ≥ 3, n (%)
Hematological toxicity			
Anemic	14(61)	3(13)	3(13)
Thrombocytopenia	13(57)	4(17)	4(17)
Leukocytopenia	11(48)	2(9)	2(9)
Neutropenia	9(39)	2(9)	2(9)
Non-hematological			
Fatigue	12(52)	0	0
Poor appetite	10(43)	0	0
Vomiting	10(43)	0	0
Nausea	7(30)	0	0
Diarrhea	6(26)	0	0
Rash	6(26)	0	0
Hyponatremia	5(22)	0	0
Increased AST level	4(17)	2(9)	0
Numbness	4(17)	0	0
Fever	4(17)	0	0
Pigmentation	4(17)	0	0
Constipation	3(13)	0	0
Increased ALT level	2(9)	0	0
Insomnia	2(9)	0	0
Increased total bilirubin	1(4)	0	0
Hypopotassemia	1(4)	0	0
Toxic epidermal necrolysis	0	1(4)	1(4)

### Association between CA19-9 decline and tumor response

**Fig. 3:**
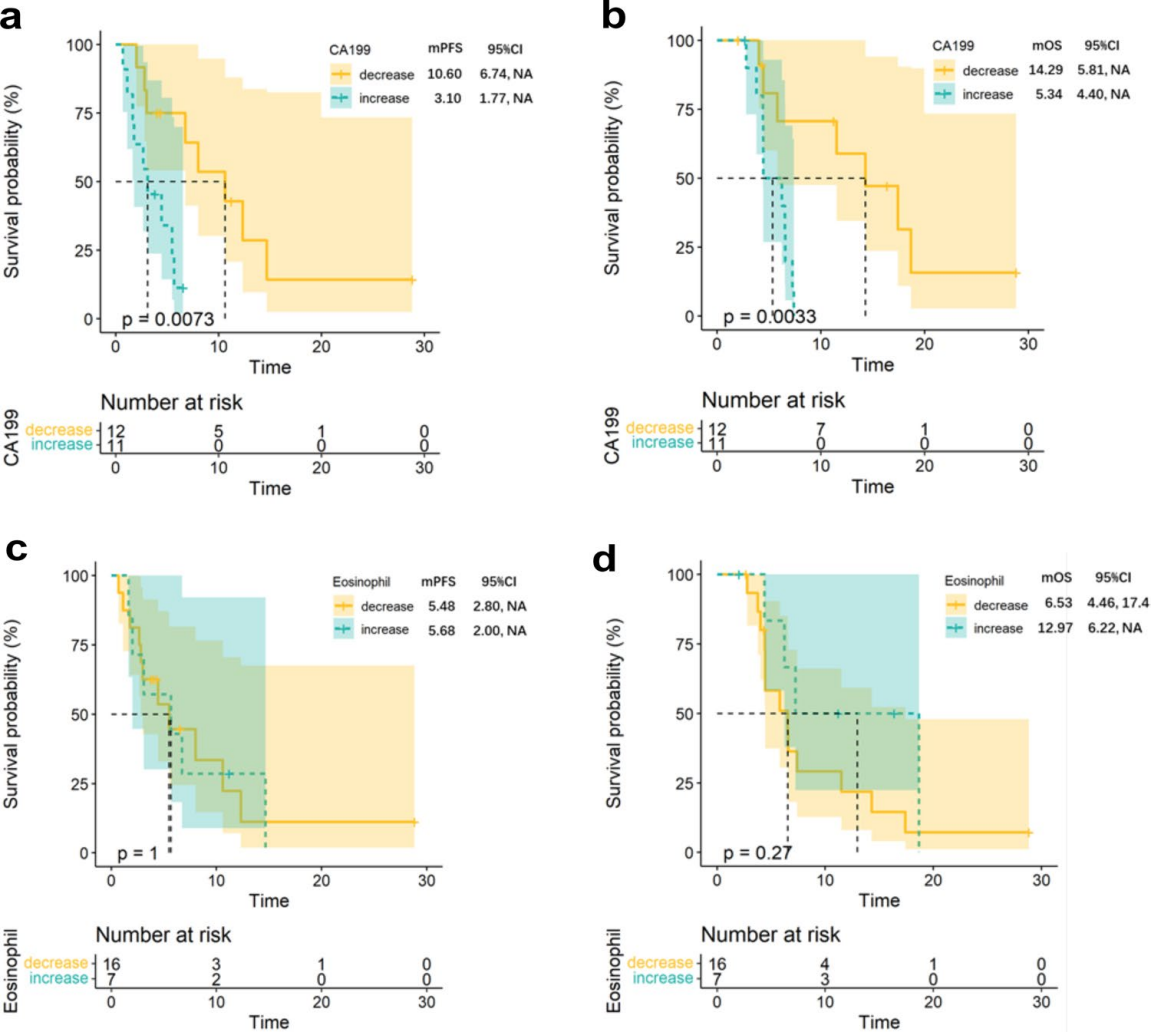
Association between peripheral blood biomarkers and treatment response **A** and **B** The Kaplan–Meier curves of **A** PFS and **B** OS of patients stratified by CA19-9 change between baseline and optimal efficacy assessment time (decline vs. elevated). **C** and **D** The Kaplan–Meier curves of **C** PFS and **D** OS of patients stratified by PBEC (declined vs. elevated). (E and F) The Kaplan–Meier curves of **E** PFS and **F** OS of patients stratified by Neutrophil (declined vs. elevated). **G** and **H** The Kaplan–Meier curves of **G** PFS and **H** OS of patients stratified by NK cell (declined vs. elevated). **I** and **J** The Kaplan–Meier curves of **I** PFS and **J** OS of patients stratified by CD3 (declined vs. elevated). **K** and **L** The Kaplan–Meier curves of **K** PFS and **L** OS of patients stratified by CD4/CD8 (declined vs. elevated)

CA19-9 is a widely recognized biomarker because of its predictive value and ability to indicate abnormal glycosylation in pancreatic cancer [33]. Notably, a normal baseline CA19-9 level and decreased CA19-9 level after treatment is associated with prolonged survival in pancreatic cancer patients. In our own investigation, we confirmed that changes in the baseline and optimal efficacy assessment times of CA19-9 levels were associated with improved overall survival and progression-free survival rates. Specifically, patients with a decrease in CA19-9 reached an mOS of 14.29 vs. 5.34 months in patients without a decrease in CA19-9 (*p* = 0.0033), and patients with a decrease in CA19-9 reached an mPFS of 10.6 vs. 3.1 months in patients without a decrease in CA19-9 (*p* = 0.0073) (Fig. 3a-b).

### Associations between peripheral blood biomarkers and tumor response

In clinical settings, the primary biomarkers used to forecast the effectiveness of immunotherapy typically include the expression of PD-L1, the level of TMB, and the status of MSI [34]. In our study, we performed immunofluorescence histochemical (IHC) analysis at the protein level on tumor biopsies from 7 patients before treatment, and one of them had biopsy after treatment due to insufficient tumor tissue obtained from biopsies. All of these 7 patients were PD-L1 negative and one patient showed high expression of PD-L1 after 3 cycles of immunotherapy combined chemoradiotherapy [35]. Given the relatively low expression levels of PD-L1 in our study group, we refrained from exploring its correlation with tumor response.

In the prespecified exploratory analysis, we assessed the correlation between clinical response and peripheral blood biomarkers that are known to be associated with the response to immunotherapy. Reportedly, PBEC is associated with a better response to immunotherapy for metastatic triple-negative breast cancer [36]. Therefore, we sought to investigate the relationship between alterations in PBEC during treatment and the clinical response. Indeed, changes in PBEC during treatment were associated with a better OS trend; however, this difference was not statistically significant. The mOS was 12.97 months for patients with elevated PBECs and 6.53 months for patients with decreased PBECs (*p* = 0.27; Fig. 3c-d). We hypothesized that the limited sample size in our study may have influenced predictive ability. Therefore, further research with larger samples is required to strengthen our findings. Moreover, we also investigate the relationship between alterations in Neutrophil, NK cell, CD3 + T-cell, and CD4/CD8 ratio during treatment and the clinical response. None of these biomarkers can help to estimate the efficacy of the therapy (Fig. S1a-h).

### Associations between pretreatment inflammatory markers and tumor response

**Fig. 4:**
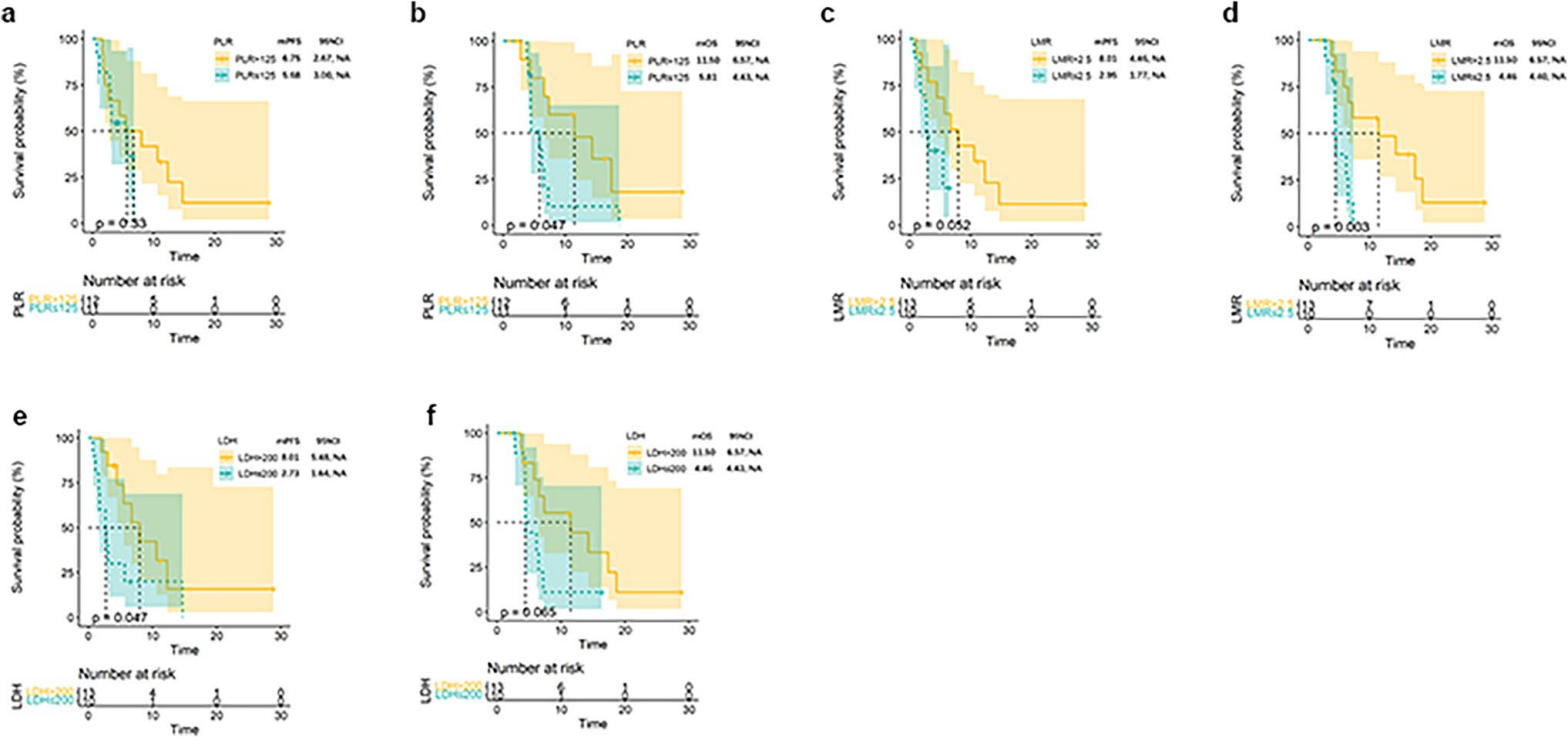
Kaplan–Meier analysis of survival and pretreatment inflammatory markers. PFS and OS based on PLR **A**-**B**, LMR **C**-**D**, and LDH level **E**–F. Abbreviations: *PFS* progression-free survival, *OS* overall survival, *PLR* platelet-to-lymphocyte ratio, *LMR* lymphocyte-to-monocyte ratio, *LDH* lactate dehydrogenase, *HR* hazard ratio, *CI* confidence interval

In our study, analysis of the relationship between inflammatory markers in peripheral blood and treatment response was conducted. A higher ORR was observed in patients with PLR > 125 (vs PLR ≤ 125, *P* = 0.0237), LMR > 2.5 (vs LMR ≤ 2.5, *P* = 0.0075); however, the ORR in these groups was not statistically significant. Furthermore, a higher DCR (92.3.0% vs. 40%, P = 0.0186) was observed in patients with LDH > 200. However, the ORR in this group did not reach statistical significance (Table 2). Patients with LDH-H (LDH > 200) had superior mPFS than those with LDH-L (LDH ≤ 200) (mPFS 8.01 vs. 2.73 months, *P* = 0.047) (Fig. 4e). Patients with PLR-H (PLR > 125) and LMR-H (LMR > 2.5) had superior mOS than those with PLR-L (PLR ≤ 125) (mOS 11.5 vs. 5.81 months, *P* = 0.047) and LMR-L (LMR ≤ 2.5) (mOS 11.5 vs. 4.46 months, *P* = 0.003) (Fig. 4b, d).

## Discussion

Pancreatic cancer is a difficult disease to treat, with low survival rates and reduced quality of life due to internal invasion and related complications. Almost all PC patients exhibit disease progression within a few months after receiving traditional treatments [37]. Surgical resection can improve patients’ long-term survival, but only a few patients can undergo surgical resection, about 50% of whom eventually experience tumor recurrence even after receiving adjuvant chemotherapy [38]. Thus, PC is expected to become the second most common cause of cancer-related death by 2030 [39]. Recently, there have been dramatic improvements in preoperative therapy for pancreatic cancer through the combination of chemoimmunotherapy with concurrent radiotherapy. However, for advanced pancreatic cancer refractory to first-line therapy, effective treatment therapy is lacking, with an average ORR of 14%. Hence, new treatments for refractory pancreatic cancer are urgently needed to improve survival.

Immunotherapy has shown remarkable treatment efficacy in numerous cancers. However, due to inherent genetic mutations and the immunosuppressive environment, immunotherapy alone or combined with chemotherapy for pancreatic cancer has been disappointing [10]. In a phase 2 study conducted by Philip Agop Philip, the ORR was 3.1% in patients treated with combination immuno-oncology therapy (anti–programmed death–ligand 1 and anticytotoxic T-lymphocyte–associated antigen 4) [40]. Recently, the emergence of immunotherapy has led to increased attention focused on radiotherapy due to its ability to induce an immune response in addition to its cytotoxic effects [41]. To date, the optimal dose and fraction of radiotherapy for enhancing the effectiveness of PD-1/PD-L1 inhibitors remains undetermined. Preclinical studies revealed that radiation doses exceeding 5 Gy per fraction can generate in situ vaccination effects, improve T-cell infiltration, and increase PD-L1 expression [42, 43]. However, a higher single dose per fraction, 15 Gy per fraction, can inhibit the antitumor response and suppress the immune environment, yielding results associated with poor prognosis [44]. Preclinical and current clinical data suggest that SBRT at a dose of 8 Gy may act as a potent immunomodulator in advanced NSCLC. This treatment modality has the potential to enhance the immune response facilitated by immune checkpoint inhibitors as evidenced by recent studies [45–48]. The increased infiltration of lymphocytes can result in the transformation of tumors into inflamed tissues that are highly receptive to T-cell activation and assault. Thus, in our study, we chose an 8 Gy*3f radiation regimen for combination therapy. Regrettably, because insufficient tissue specimens were obtained before or after SBRT, we were unable to explore the impact of radiation therapy on the tumor microenvironment in these individuals. In addition, highly effective responses to immunotherapy can result in decreased tumor burden [49]. Chemotherapy can also induce immunogenic death of tumor cells and further enhance immunotherapy effects. The decreased tumor burden induced by RT and chemotherapy may contribute to enhanced responses to subsequent PD-1 antibody immunotherapy. Thus, combining immunotherapy, hypofractionated radiotherapy, and chemotherapy might be a promising approach for treating refractory metastatic pancreatic cancer patients.

Our study suggested that the combined approach of hypofractionated radiotherapy, anti-PD-1 antibody therapy, and chemotherapy provides noteworthy antitumor effectiveness and causes durable clinical response. In a phase 2 clinical trial, the clinical benefits and safety of nivolumab with or without ipilimumab in combination with stereotactic body radiotherapy (SBRT) in patients with refractory mPC were evaluated. The ORR and DCR in nivolumab with ipilimumab in combination with stereotactic body radiotherapy (SBRT) treatment group. The median PFS and OS were 1.6 (95% CI, 1.6 to 2.8) and 3.8 months (95% CI, 2.8 to 6.5), respectively [30]. Yoo and colleagues [50] did a randomized phase 2 trial comparing modified versions of FOLFOX (folinic acid, fluorouracil, and oxaliplatin) and FOLFIRI (folinic acid, fluorouracil, and irinotecan) regimens for treatment of gemcitabine-refractory advanced pancreatic cancer. However, in that study, the median overall survival was short (3·5 months with FOLFOX and 3·9 months with FOLFIRI). Moreover, in a global, phase 3, randomized clinical trial, the median OS and PFS in patients assigned nanoliposomal irinotecan plus fluorouracil and folinic acid was 6·1 months (95% CI 4·8–8·9) and 4·9 months [4·2–5·6]. Besides, The ORR was 16%. [5] The ORR and DCR of our study were 69.5% and 30.4%, respectively, which were greater than those of previously reported clinical trials. In addition, the mPFS of our study was 5.48 months, which is greater than the 4.9 months reported for NAPOLI-1. Notably, three patients did not reach mPFS at the last follow-up (August 30, 2023), and two patients had PFS greater than 1 year.

To our knowledge, decreased CA19-9 was associated with superior survival outcomes and clinical response in pancreatic cancer patients who received a preoperative treatment regimen of PD-1 blockade plus chemoradiotherapy, providing a viable predictive biomarker [32]. In our study, we also observed a significant association between decreased CA19-9 and prolonged PFS or OS in pancreatic cancer patients who received second-line therapy, which means that CA19-9 might be a viable predictive biomarker for second-line treatment. Early studies on ICIs have established a positive association between increased PBEC and improved survival rates in patients with melanoma [36]. In addition, an increased PBEC after ICI treatment has a positive correlation with a superior response to ICIs in several types of cancer [51, 52]. Eosinophils can infiltrate tumors and directly interact with tumor cells or indirectly reshape the tumor immune microenvironment [53]. However, the association between an increase in the PBEC and a superior response to ICIs in pancreatic cancer patients has not been reported. Thus, in our research, we observed that patients with elevated PBECs exhibited better OS trends, but the difference was not statistically significant, which might be due to the limited sample size.

Recently, inflammatory response factors, such as NLR, PLR, LMR, and LDH were found to be associated with tumor response and patients’ prognosis. In a retrospective study of peripheral blood markers predictive of the outcome in patients with advanced pancreatic cancer who underwent anti-PD-1 therapy, they found that patients had baseline NLR ≤ 2 achieved higher ORR and DCR, and patients with PLR ≤ 135, LMR > 2, and LDH ≤ 265 could also observed a higher DCR [54]. Given the crucial role of inflammation in the initiation, promotion, and advancement of cancer, we undertook analysis to investigate the link between peripheral blood inflammatory markers and clinical response. Intriguingly, we discovered that the initial PLR, LMR, and LDH level could forecast tumor response. In agreement with previous findings, higher LMR of our study cohort proved to be associated with higher ORR and mOS. Moreover, it should be emphasized that no clear cutoff values of the LMR have been agreed on. When examining our study cohort, we identified the optimal cutoff value to be 2.5, which is in the range of and in very good agreement with previously reported values varying from 2.05 to 4.62 [55]. Another inflammatory-based marker to be considered is represented by the PLR. In a meta-analysis of 17 cohorts, a low PLR was linked to longer mOS (HR = 1.28, 95% CI = 1.17–1.40, *p* = 0.00001). Cutoff values ranged from 126 to 300 [56]. In our study cohort, higher PLR was associated with superior mOS and higher ORR. We also found that pretreatment LDH levels appeared to be associated with DCR and OS, which did not align with previous reported data[57].

There are several limitations that should be acknowledged when interpreting our study. First, the small sample size and limited data may not accurately represent a broader population of pancreatic patients. Second, due to the invasive nature of sampling, we were unable to acquire tumor tissue before and after integrated immunotherapy. Hence, we could not investigate whether MSI/dMMR status and PD-L1 expression were altered following this treatment. As such, the association between these biomarkers and the benefits of our therapy could not be analyzed. Third, our study did not include a control group, and we only compared our results with data reported in previous studies. Finally, further research is required to elucidate the underlying mechanisms of our treatment.

In summary, this is a prospective clinical trial in which a regimen of chemoimmunotherapy concurrent with radiotherapy was adopted for patients with pancreatic cancer. These findings demonstrate notable potential for improving treatment efficacy in mPC patients after failure of first-line chemotherapy. Besides, the pretreatment peripheral blood marker NLR and decreased CA199 level during treatment might correlate with tumor response in patients treated with this regime.

## Supplementary Information

Below is the link to the electronic supplementary material.

Supplementary file1 (DOCX 14 KB)

Supplementary file2 (TIF 7058 KB)
