# Supplementary material for: Hypofractionated radiotherapy plus PD-1 antibody and SOX chemotherapy as second-line therapy in metastatic pancreatic cancer: a single-arm, phase II clinical trial
Source: Cancer Immunol Immunother. 2024 Aug 6;73(10):201. doi: 10.1007/s00262-024-03744-z (PMC11303639; doi:10.1007/s00262-024-03744-z)
Supplement: Supplementary file 1 — Supplementary file1 (DOCX 14 KB) [file 262_2024_3744_MOESM1_ESM.docx]

**Table 1 The inclusion and exclusion criteria of the protocol.**

| Inclusion criteria | Exclusion criteria |
| --- | --- |
| a. Subjects with age ≥18 years and ECOG score of 0–1; | a. Patients who have received more than second-line systematic anti-tumor treatment; |
| b. Subjects with histologically or cytologically proven adenocarcinoma of the pancreas; | b. Patients who have received fluorouracil, platinum, or anti-PD-1 monoclonal antibodies in first-line treatment; |
| c. The patients with at least one measurable or evaluable distant metastasis lesion based on CT or MRI scanning; | c. Patients with previous history of other tumors, except for cervical cancer in situ, treated squamous cell carcinoma or bladder epithelial tumor (TA and TIS) or other malignant tumors that have received radical treatment (at least 5 years before enrollment; |
| d. Subjects failed a gemcitabine-based palliative frontline chemotherapy, and did not receive fluorouracil, platinum, or anti-PD-1 monoclonal antibodies before; | d. Patients with active bacterial or fungal infection (≥level 2 of NCI-CTC, 3rd Edition); |
| e. The subjects should meet the following hematological indexes: Neutrophil count ≥1.5 ∗ 10^9^/L, Hemoglobin ≥10 g/dl, Platelet count ≥100 ∗ 10^9^/L; | e. Patients with HIV, HCV, HBV infection, uncontrollable coronary artery disease or asthma, uncontrollable cerebrovascular disease or other diseases considered by researchers to be out of the group; |
| f. The subjects should meet the following biochemical indicators: Total bilirubin ≤1.5 ∗ULN; AST and ALT <1.5 ∗ ULN; Creatinine clearance rate ≥60 ml/min; | f. Patients with autoimmune diseases or immune defects who are treated with immunosuppressive drugs; |
| g. Subjects of childbearing age need to take appropriate protective measures (contraceptive measures or other methods of birth control) before entering the group and during the test; | g. Pregnant and lactating women. Pregnant women of childbearing age must be tested negative within 7 days before entering the group; |
| h. Subjects who have signed informed consent; | h. Patients with drug abuse, clinical or psychological or social factors make informed consent or research implementation affected; |
| i. Subjects who were able to follow the protocol and follow-up procedures. | i. Patients who may be allergic to PD-1 monoclonal antibody immunotherapy drugs. |
