# Supplementary figures and images for: Hypofractionated radiotherapy plus PD-1 antibody and SOX chemotherapy as second-line therapy in metastatic pancreatic cancer: a single-arm, phase II clinical trial
Source: Cancer Immunol Immunother. 2024 Aug 6;73(10):201. doi: 10.1007/s00262-024-03744-z (PMC11303639; doi:10.1007/s00262-024-03744-z)

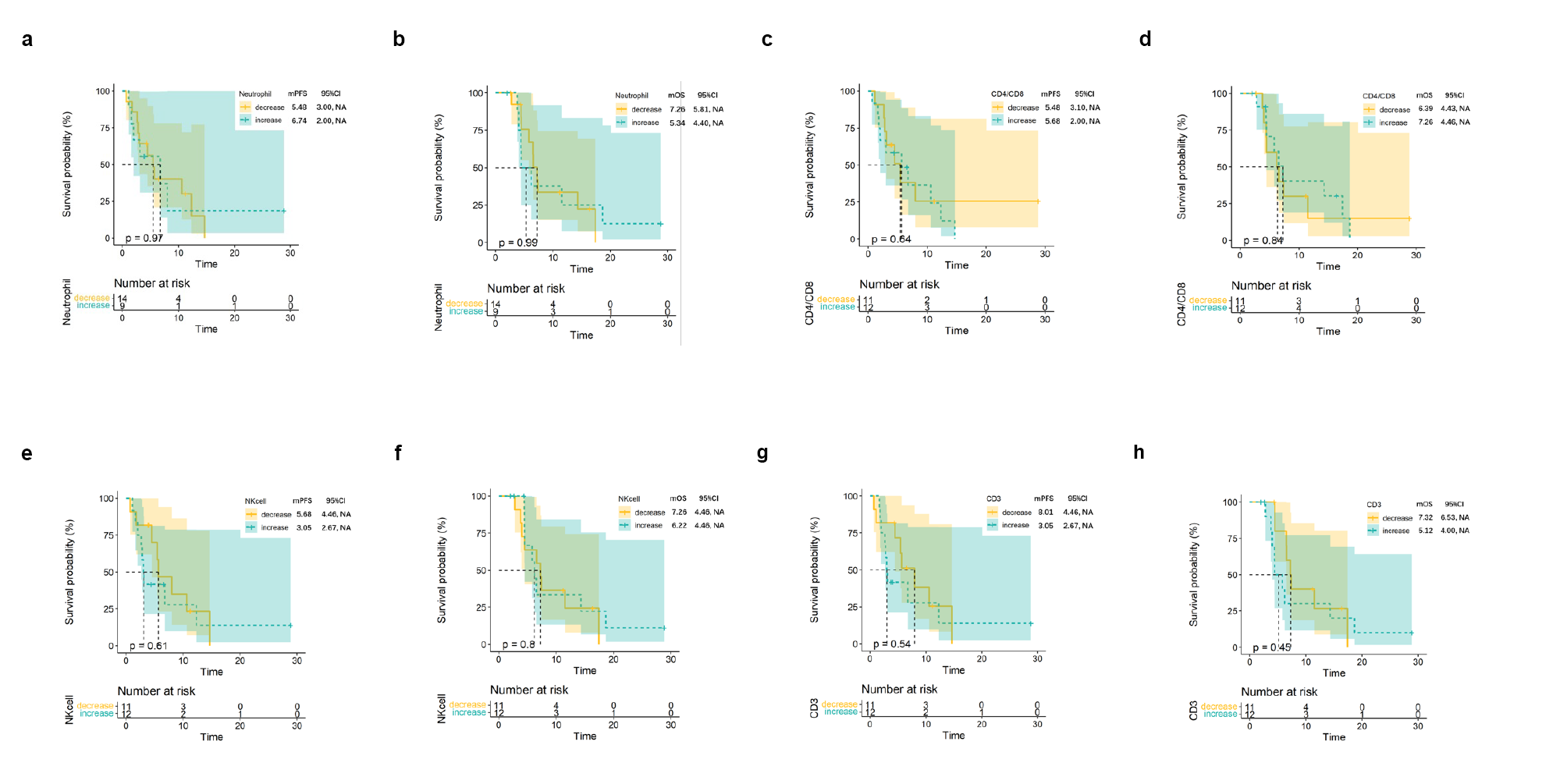

Supplement: Supplementary file 2 — Supplementary file2 (TIF 7058 KB) [file 262_2024_3744_MOESM2_ESM.tif]
